# Sorption studies of europium on cerium phosphate using Box-Behnken design

**DOI:** 10.3906/kim-2002-16

**Published:** 2020-08-18

**Authors:** Süleyman İNAN

**Affiliations:** 1 Institute of Nuclear Sciences, Ege University, İzmir Turkey

**Keywords:** Sorption, europium, cerium phosphate, Box-Behnken, separation

## Abstract

Amorphous cerium phosphate was prepared and characterized. Three-level Box-Behnken design (BBD) was employed to analyze the effect of process variables such as initial pH (2–6), contact time (60–180 min), and sorbent amount (0.05–0.15 g) on the sorption capacity of europium. Analysis of variance (ANOVA) revealed that the main effect of initial pH and sorbent amount has a substantial impact on the sorption of Eu(III). Probability F-value (F = 3 × 10^-3^) and correlation coefficient (R2 = 0.97) point out that the model is in good accordance with experimental data. The maximum sorption capacity of Eu(III) was found to be 42.14 mg g^-1^ at initial pH 6, contact time of 180 min, and a sorbent amount of 0.05 g. Sorption isotherm data was well explained by the Langmuir model and monolayer Eu(III) sorption capacity was obtained as 30.40 mg g^-1^. Kinetic data were well described by the pseudo-second-order model. Thermodynamic data suggested that the process is endothermic and spontaneous.

## 1. Introduction

Rare earth elements (REEs) are a group of metals consisting of 15 lanthanide elements including yttrium and scandium. Owing to their superior physical and chemical properties, they are significant for several progressive technologies [1]. Among them, europium (Eu) is an active metal; a trace amount of europium may greatly enhance the properties of metals [2]. Europium is used in light-emitting diodes (LEDs), LCDs, and fluorescent lamps. Because of the growing demand for LEDs and LCDs, it is becoming a more vital element for the electronic industry [3]. Europium is also used in nuclear reactor control rods because of its neutron absorption effectiveness1. The demand for europium supply has been increasing every day. However, europium sources are not equally distributed on Earth, and the production of europium in the world is mostly in China [3]. Therefore, recovery of europium and other REEs from alternative sources such as electronic waste and wastewater has become an important issue.

On the other hand, during the operation of nuclear power plants radioactive europium (^152+154^Eu) is produced in low-level radioactive waste solutions together with 137 Cs. Because of its long half-life, separation of europium from radioactive wastes before discharge is very significant [4]. Recovery and removal of europium from a different type of waste solutions are of great importance not only from the point of their limited resource availability but also for the mitigation of their quantity for disposal as radioactive wastes.

Sorption is one of the most promising techniques due to its advantages of high efficiency, low operation temperature, and high selectivity. Various types of materials such as goethite [5], aluminum silicate [6], aluminum oxide [4], functionalized magnetic chitosan [7], montmorillonites [8], manganese oxides [9], biomass [10] and algae [3] were used as sorbents/ion exchangers to remove and separate europium from radioactive waste and aqueous streams.

Insoluble salts of tetravalent metals are composed of tetravalent metal salts and anions like phosphate, molybdate, antimonate, etc. Inorganic ion exchangers/sorbents consisted of tetravalent metal acid salts are highly stable in strong acids, oxidizing solutions, and ionizing radiations. They show selectivity for certain metal ions. In particular, they are attractive for applications where organic resins cannot be used because of their degradability [11]. Cerium(IV) tungstate [12], titanium(IV) phosphates [13,14], poly-acrylamide based cerium(IV) phosphate [15], zirconium(IV) molybdate [16] have been utilized for the sorption of europium ions. Cerium phosphate compounds have been prepared [17–19] and used for the separation of some radionuclides [15,20] from hazardous waste solutions. But so far, there is no study reported in the literature focused on the sorption behavior of neat cerium phosphate towards europium.

In the present study, amorphous cerium phosphate was prepared, characterized, and used as a sorbent for the separation of Eu(III) ions from aqueous solution. Response surface methodology (RSM) was utilized to analyze the effect of initial pH, contact time, and sorbent amount on sorption capacity. For this aim, Box-Behnken Design (BBD) with 3 variables was employed. Equilibrium isotherm, kinetic and thermodynamic studies were conducted to assess the sorption behaviors.

## 2. Materials and methods

### 2.1. Reagents

Europium nitrate pentahydrate (Eu(NO_3_)_3_•5H_2_O) was purchased from Sigma-Aldrich Corp. (St. Louis, MO, USA). Cerium(IV) sulfate tetrahydrate (Ce(SO_4_)_2_•4H_2_O), dipotassium hydrogen phosphate (K_2_HPO_4_), ammonia solution and nitric acid were obtained from Merck. The stock solution of Eu(III) (1000 mg L^-1^) was prepared by dissolving 2.82 g of Eu(NO_3_)_3_•5H_2_O in 1000 mL deionized water. Test solutions of the chosen concentrations were prepared by diluting the stock solution in appropriate volumes. Nitric acid and ammonia solution were used for pH adjustments.

### 2.2. Preparation of cerium phosphate

The precipitation of amorphous cerium phosphate was reported by Hanna et al. [21]. According to this procedure, the stoichiometric amount of Ce(SO_4_)_2_•4H_2_O and K_2_HPO_4_ was dissolved in deionized water and equimolar (0.012 mol L^-1^) solutions of Ce(SO_4_)_2_•4H_2_O and K_2_HPO_4_ were prepared separately. K_2_HPO_4_ solution was added to Ce(SO_4_)_2_•4H_2_O solution dropwise with continuous stirring until the final pH of the solution was 2. The mixture obtained was stirred for 1 h more and kept 24 h at ambient temperature for aging. The yellow-colored precipitate was washed with deionized water to obtain a constant pH value. The formed gel was separated by centrifuge and dried at 60 ° C.

### 2.3. Identification and characterization

Cerium phosphate powders were identified and characterized by X-ray Diffraction (XRD), scanning electron microscope (SEM), Fourier transform infrared (FTIR), and thermal analyses (TGA-DSC). XRD pattern was recorded by Philips X’Pert Pro diffractometer (Philips Research Laboratories, Eindhoven, Netherlands). FTIR data (400-4000 cm^-1^) were acquired by Perkin Elmer Spectrum Two model FTIR-ATR spectrometer (PerkinElmer, Inc. Waltham, MA USA). SEM images were collected using the Thermo Scientific Apreo S model scanning electron microscope (Thermo Fisher Scientific Inc., Waltham, MA, USA). TGA-DSC data were obtained by TA Instruments SDT Q600 up to 1000 ° C with a heating rate of 10 ° C min^-1^ (TA Instruments SDT Q600: TA Instruments, New Castle, DE, USA).

### 2.4. Statistical design of experiments

Response surface methodology (RSM) is a useful tool to evaluate the interactions of process variables [22]. RSM is composed of statistical and mathematical techniques to design experiments, establish numerical models, study the effects of variables, and search for the optimum combinations of factors. In experimental design methodology, variables vary from one experiment to the next simultaneously. As the variables may affect each other, the value of one variable can depend on the value of other variables [23].

Three-level designs have been suggested by Box and Behnken (1960) for fitting the response surfaces. In these designs, 2k factorials are combined with incomplete block designs. The resulting designs are composed of a reduced number of required runs which make them efficient [24].

To investigate the effect of initial pH (X_1_), contact time (X_2_), and sorbent amount (X_3_) on Eu(III) sorption, a three-level BBD has been used. According to BBD, a total of 15 runs with three replicates at center points were carried out. Statistical analyses were performed by Design Expert 12 software (Stat-Ease, Inc., Minneapolis, MN, USA). The effect of variables on Eu(III) sorption was investigated by batch method. The contact between sorbent and Eu(III) ions in solution was provided in a temperature-controlled shaker at 150 rpm by adding 0.05 g sorbent in 30 mL of liquid phase. When the sorption equilibrium was achieved, samples were filtered and Eu(III) concentrations were determined by ICP-OES.

Sorption capacity (Q) was calculated using Eq. (1):

(1)Q=(C0-Ce)xVm(mgg-1)

Distribution coefficient ( *K_D_* ) is defined as the ratio of metal concentration in the sorbent phase to solution-phase [25] and it is determined by Eq. (2):

(2)KD=C0-CeCeXVm(mLg-1)

In Eqs. (1) and (2), Co and Ce are the initial and equilibrium concentrations of Eu(III) ion in solution (mg L^-1^), *V* is the volume (mL) and m is the mass of the sorbent (g).

The ranges and levels of the variables (low, center, high) are presented in Table 1.

**Table 1:** Range and levels of process variables.

Variable		-1	0	+1
Initial pH	X_1_	2	4	6
Contact time (min)	X_2_	60	120	180
Sorbent amount (g)	X_3_	0.05	0.10	0.15

The general model equation including linear and quadratic terms for the estimation of optimum response is as follows:

(3)yi=b0+biXi+biiXii2bijXiXj

In Eq. (2), bo denotes the intercept. bi , bii and bij signify linear effects, quadratic effects and dual effects, respectively. The second-order polynomial equation related to the model can be written as follows:

(4)y=b0+b1X1+b2X2+b3X3+b11X12+b22X22+b33X32+b12X1X2+b13X1X3+b23X2X3

### 2.5. Isotherm, kinetic and thermodynamic studies

#### 2.5.1. Isotherm studies

30 mL of Eu(III) solutions at varying concentrations (25–400 mg L^-1^) were separately prepared. After adjusting the pH of the solution to 4, 0.05 g cerium phosphate was added to each solution. The solid/liquid contact was carried out in a temperature-controlled shaker at 298 ± 1 K for 120 min.

#### 2.5.2. Kinetic studies

Kinetic experiments were conducted between 15 and 360 min of contact time. 30 mL of 100 mg L^-1^ Eu(III) solutions were separately contacted with 0.05 g sorbent at pH 4. Kinetic models were applied to test the experimental data.

#### 2.5.3. Thermodynamic studies

Experiments were carried out at 303, 313, 323, and 333 K for the determination of thermodynamic parameters such as enthalpy change (ΔH°) , entropy change (ΔS°) and Gibbs free energy change (ΔGo) . For each temperature, 30 mL of 100 mg L^-1^ Eu(III) solution was shaken with 0.05 g of sorbent at pH 4 for 120 min.

For all experiments, initial and equilibrium concentration of Eu(III) ions were measured by ICP-OES. Eu(III) sorption capacity (Q) and distribution coefficient values (KD) were calculated according to Eqs. (1) and (2), respectively. 

## 3. Results and discussion

### 3.1. Characterization studies

XRD pattern of cerium phosphate is depicted in Figure 1a. It is seen that cerium phosphate has an amorphous structure. Figure 1b represents the IR spectrum of the cerium phosphate powders. The broad peak at 3196 cm^-1^ is assigned to –OH stretching vibrations and the peak at 1633 cm^-1^ is attributed to –OH bending vibrations of water molecule [26]. The bands around 992 cm^-1^ and 513 cm^-1^ correspond to P–O stretching and O–P–O bending mode of vibration, respectively [21].

**Figure:**
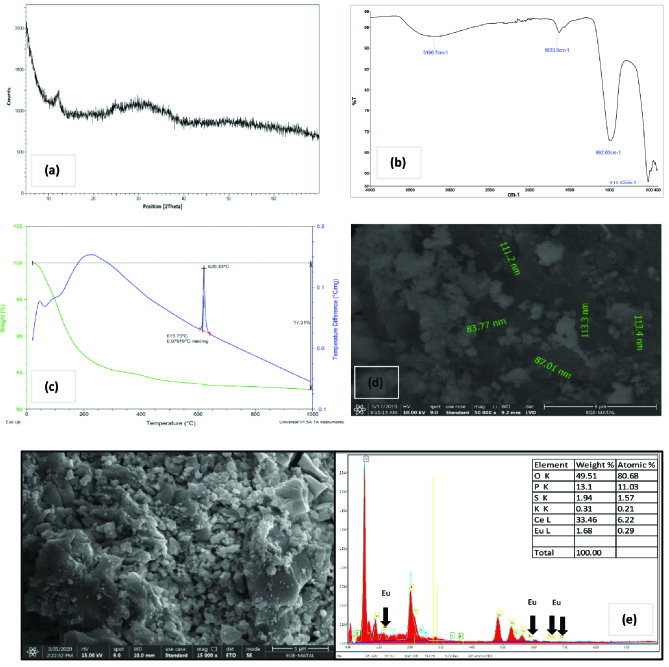
Characterization studies; a) XRD pattern, b) IR spectrum, c) TGA-DSC curve, d) SEM image before Eu(III) sorption, e) SEM image & EDX data after Eu(III) sorption.

TGA-DSC thermogram of cerium phosphate is demonstrated in Figure 1c. The thermogram was taken between 10 ° C and 1000 ° C at a uniform heating rate of 10 ° C min^-1^. The TGA curve shows a continuous decrease in the mass as a function of temperature. There are two weight loss regions. The weight loss between 25 ° C and 310 ° C, is mainly from the dehydration of physisorbed water molecules. Condensation of POH groups occurs from 310 ° C to 1000 ° C. The total weight loss is about 17.31%. In the DSC curve, the endothermic peak around 75 ° C is attributed to the dehydration of water. The sharp exothermic peak at 620.33 ° C is due to the crystallization of cerium polyphosphate. SEM image of cerium phosphate powders is given in Figure 1d. Particles tend to form aggregates. Smaller particles with an approximate size of 80 to 120 nm were detected.SEM image and EDX data after Eu(III) sorption are presented in Figure 1e. There is no significant change in the SEM image after sorption. Particle size distribution is not homogeneous and aggregates can be seen. The presence of Eu(III) on the sorbent surface was confirmed by EDX data. The weight percentage of elements on the surface was also provided in Figure 1e.

### 3.2. Statistical analysis

BBD utilized in this study consists of 12 factorial and 3 replicate points. Experimental variables in coded and actual forms along with the response are presented in Table 2.

**Table 2:** Box-Behnken model for Eu(III) sorption onto cerium phosphate.

No	Initial pH	Contact time (min)	Sorbent amount (g)	Experimental Eu(III) capacity (mg g^-1^)	Predicted Eu(III) capacity (mg g^-1^)
1	- 1(2)	- 1(60)	0(0.10)	7.39	6.06
2	1(6)	- 1(60)	0(0.10)	15.55	18.46
3	- 1(2)	1(180)	0(0.10)	9.09	6.18
4	1(6)	1(180)	0(0.10)	23.05	24.39
5	- 1(2)	0(120)	- 1(0.05)	9.59	11.42
6	1(6)	0(120)	-1(0.05)	41.87	39.44
7	- 1(2)	0(120)	1(0.15)	6.15	8.57
8	1(6)	0(120)	1(0.15)	12.98	11.16
9	0(4)	- 1(60)	- 1(0.05)	25.37	24.88
10	0(4)	1(180)	- 1(0.05)	27.19	28.28
11	0(4)	- 1(60)	1(0.15)	10.77	9.69
12	0(4)	1(180)	1(0.15)	11.85	12.34
13	0(4)	0(120)	0(0.10)	15.80	15.82
14	0(4)	0(120)	0(0.10)	15.66	15.82
15	0(4)	0(120)	0(0.10)	16.02	15.82

A second-order polynomial equation was used to express the relationship between independent variables and the response. The regression coefficients of parameters were determined and data were fitted to a secondorder polynomial equation as given by Eq. (5):

(5)y=15.82+7.65XX1+1.51X2-7.78X3-1.60X12-0.45X22+3.42X32+1.45X1X2-6.36X1X3-0.19X2X3

The statistical significance of the model is checked by ANOVA [27]. Table 3 represents the ANOVA results, coefficients, and P values. In Table 3, the model F value of 15.93 suggests that the proposed model is significant. The correlation coefficient (R2) was found to be 0.97. It means that predicted capacity values are in good agreement with the experimental ones.

**Table 3:** ANOVA, coefficients and P values for Eu(III) sorption onto cerium phosphate

ANOVA.						
	df	Sum of squares	Mean square	F value	Probability F	R^2^
Regression	9	1199.432	133.270	15.927	0.004	0.97
Residuals	5	41.839	8.368			
Total	14	1241.271				
	Coefficients	P values				
Intercept	15.82	0.0002				
X_1_	7.65	0.0007				
X_2_	1.51	0.1992				
X_3_	− 7.78	0.0006				
X_1_X_1_	− 1.60	0.3360				
X_2_X_2_	− 0.45	0.7751				
X_3_X_3_	3.42	0.0720				
X_1_X_2_	1.45	0.3612				
X_1_X_3_	− 6.36	0.0070				
X_2_X_3_	− 0.19	0.9020				

The smallest level of importance leading to rejection of the null hypothesis is described as the P-value. When P < 0.05, the main effect and dual effects are regarded as statistically significant [28]. The main effectof initial pH (P = 7.0 × 10^-4^), the main effect of sorbent amount (P = 6.0 × 10^-4^), and dual effect of initial pH and sorbent amount (P = 7.0 × 10^-3^) were determined to be significant.

**Figure 2:**
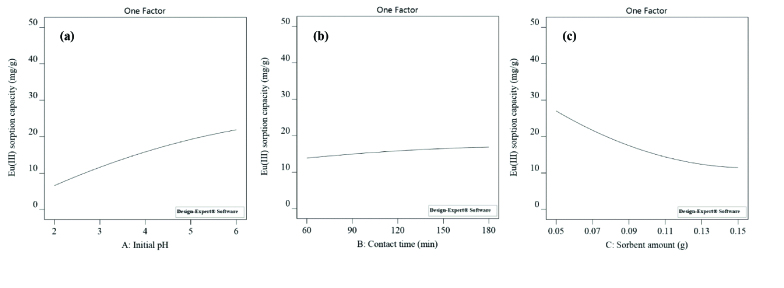
Main effects on Eu(III) sorption capacity; a) effect of initial pH, b) effect of contact time, c) effect of sorbent amount.

The positive value of coefficient belonging to pH (X_1_ = 7.65) points out that pH has a positive effect onthe sorption of Eu(III). It means that the uptake of Eu(III) increases as pH increases. The sorbent surface is mostly protonated at lower pHs. Eu(III) ions in solution compete with H^+^ ions for the active surface sites. As the pH increases, the number of H+ ions in the solution decreases, and the sorption of Eu(III) becomes more favorable [29]. It is seen from Figure 2a that the sorption capacity of Eu(III) increases in the pH range of 2−6. Maximum Eu(III) uptake is 21.87 mg g^-1^ at pH 6. Contact time (X_2_ = 1.51) has a positive effect on sorption.Eu(III) sorption capacity slightly increases from 13.85 to 16.88 mg g^-1^ (Figure 2b).

However, the coefficient of sorbent amount (X_3_ = − 7.78) has a negative value. This implies that the sorption capacity of Eu(III) decreases with the increasing sorbent amount. Eu(III) uptake decreased from 27.03 to 11.46 mg g^-1^ with the increase in sorbent amount from 0.05 to 0.15 g (Figure 2c).

### 3.3. 3D Response surface plots

**Figure 3:**
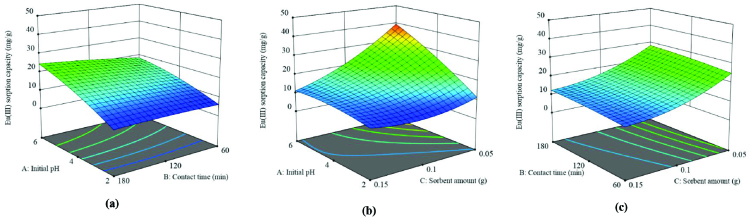
Response surface plots for dual effects; a) initial pH and contact time, b) initial pH and sorbent amount, c) contact time and sorbent amount.

The relation between the dual effects of independent variables and Eu(III) sorption capacity (Q) was given in 3D surface plots (Figure 3). Figure 3a displays the change in Q values depending on initial pH (X_1_) and contact time (X_2_) . Maximum Q was found to be 24.39 mg g^-1^ in 180 min, at initial pH 6 by holding the sorbent amount at 0.10 g.

Figure 3b shows the dual effect of initial pH (X1) and sorbent amount (X_3_). Maximum Q was 39.44 mg g^-1^ at initial pH 6 and sorbent amount of 0.05 g. The dual effect of contact time (X_2_) and sorbent amount (X_3_) at the initial pH 4 is shown in Figure 3c. It is inferred from Figure 3c that Q value gradually increases with the increase in contact time from 60 to 180 min. However, a significant decrease can be seen on the Q values by the increase in sorbent amount in the range of 0.05–0.15 g. Maximum Q was determined to be 28.28 mg g^-1^ in 180 min with 0.05 g sorbent.

### 3.4. Equilibrium isotherm, kinetic and thermodynamic studies

#### 3.4.1. Isotherm studies

**Figure 4:**
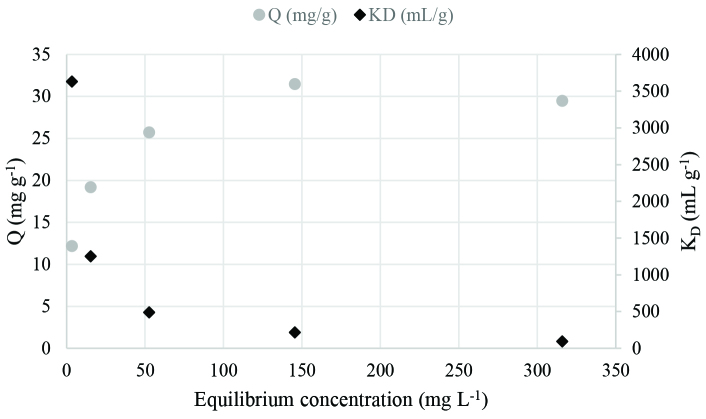
Sorption isotherm of Eu(III) onto cerium phosphate (initial pH: 4, temperature: 25 °C, contact time: 120 min).

The effect of initial Eu(III) concentration on sorption was determined between 25–400 mg L^-1^. Figure 4 demonstrates the relation between Q, K_D_, and Eu(III) ion concentration at equilibrium. Eu(III) uptake increases from 12.17 to 29.47 mg g^-1^ with the increase in initial Eu(III) concentration from 25 to 200 mg L^-1^. After this point, Eu(III) uptake reaches a plateau and remains almost constant. This behavior can be interpreted as the saturation of active sites on the sorbent. On the other hand, with the increase in initial Eu(III) concentration, K_D_ values decrease since the number of ions in the solution increases more than the number of ions sorbed. The maximum K_D_ value of 3632.2 mL g^-1^ was obtained when the initial Eu(III) concentration was 23.6 mg L^-1^.

The sorption equilibrium data are expressed by sorption isotherms. Isotherms clarify the relationship between the metal concentration in the solution at equilibrium Ce and the mass of the metal sorbed per unit mass of sorbent qe .

Langmuir’s theory assumes that sorption occurs at specific homogeneous sites within the sorbent. Langmuir model in its linear form [30] is expressed as:

(6)Ceqe=1qmb+Ceqm

where q_m_ is the maximum amount of the metal ion per unit weight of adsorbent to form a complete monolayer (mg g^-1^), C_e_ is the equilibrium concentration of metal ion (mg L^-1^) and b is a constant related to the sorption energy (L mg^-1^). q_m_ and b values are calculated from the slope and intercept of the linear plot between C_e_/q_e_ and C_e_.

q_m_, b, and correlation coefficient (R^2^) values calculated from the isotherm are given in Table 4. qm value was estimated to be 30.40 mg g^-1^. An R^2^ value of 0.99 indicates that the sorption data can be explained well by the Langmuir model.

**Table 4:** Isotherm model data for Eu(III) sorption onto cerium phosphate.

Isotherm model	Parameter	Value
Langmuir	qm (mg g^-1^) 30.40	30.40
	b (L mg^-1^)	0.18
	R^2^	0.99
Freundlich	K_f_	10.3
	n	4.83
	R^2^	0.92
Dubinin–Radushkevich (D–R)	qm (mg g^-1^)	26.44
	E (kj mol^-1^)	0.50
	R^2^	0.80

**Figure 5:**
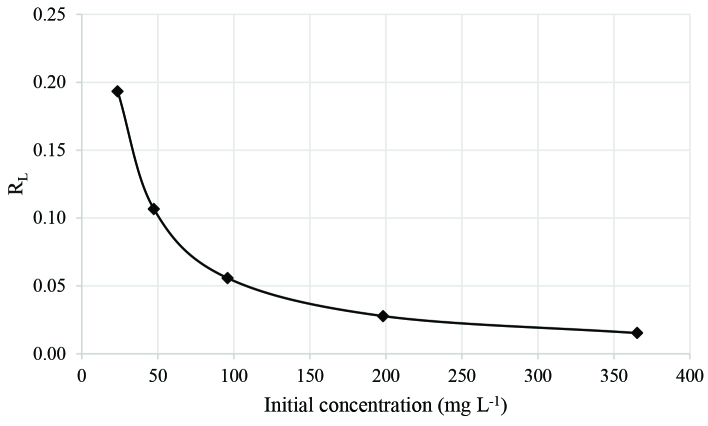
Dimensionless constant (R_L_).

The separation factor (R_L_) [31], which is based on the Langmuir model, can be represented as in Eq. (7):

(7)RL=11+bC0

where b is the Langmuir constant (L mg^-1^) and C_0_ is the initial metal ion concentration (mg L^-1^). If the value of R_L_ is between zero and one (0 < RL < 1), the sorption is favorable. The calculated values of R_L_ were plotted against initial Eu(III) concentration as shown in Figure 5. It can be deduced from Figure 5 that Eu(III) sorption is favorable for all concentrations under investigation.

Freundlich adsorption isotherm [32] and its linear form can be described by Eq. (8) and Eq. (9), respectively:

(8)qe0KfCe1n

(9)lnqe=lnKf+1nlnCe

where q_e_ is the equilibrium adsorption capacity (mg g^-1^), C_e_ is the metal concentration at equilibrium, K_f_ and n are constants related to adsorption capacity and intensity, respectively. The values of n and K_f_ are shown in Table 4.

Dubinin-Radushkevich isotherm [33] is expressed as:

(10)lnqe=lnqm-βε2

ε is the Polanyi potential given by Eq. (11),

(11)ε=RTln(1+1Ce)

where β is a constant related to the mean free energy of adsorption (mol^2^J^2^)^-1^, q_e_ is the amount of solute adsorbed at equilibrium (mg g^-1^), q_m_ is the theoretical saturation capacity (mg g^-1^), R is the gas constant (R = 8.314 J mol^-1^K^-1^) and T is the temperature (K). The adsorption mean free energy E (kJ mol^-1^) can be calculated as follows:

(12)E=12β

When the value of E is below 8 kJ mol^-1^, the sorption process can be considered as physical sorption. However, the sorption process follows a chemical mechanism if the value of E is between 8–16 kJ mol^-1^. The D-R isotherm constant and the value of E are summarized in Table 4. The adsorption mean energy value was obtained as 0.50 kJ mol^-1^. This value indicates that physical sorption plays a dominant role in the sorption process.

The comparison of Eu(III) sorption capacity of amorphous cerium phosphate with some other sorbents reported in the literature is presented in Table 5.

**Table 5:** Comparison of Eu(III) sorption capacities of various sorbents.

Sorbent	Initial pH	Eu(III) Capacity (mg g^-1^)	Reference
Poly-acrylamide based Ce(IV) phosphate	2.0	91.2	[15]
Aluminum silicotitanate	4.0	45.9	[34]
Al^3+^ and Fe^3+^ modified titanium phosphates	6.0	39.0	[35]
Magnesia modified aluminum silicate	6.0	48.9	[6]
Al-substituted goethite	5.5	6.75	[5]
Cerium phosphate	6.0	42.14	Present study

As can be seen in Table 5, the obtained Eu(III) uptake value of cerium phosphate is comparable to the values reported in the literature. On the other hand, the uptake capacity of cerium phosphate can be enhanced by surface modification or doping of other elements.

#### 3.4.2. Thermodynamic studies

**Figure 6:**
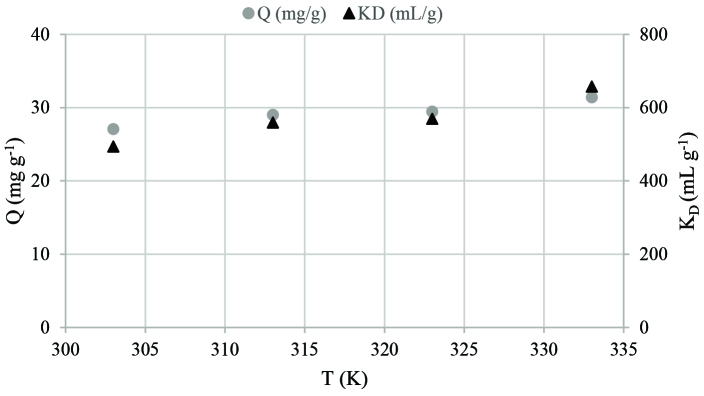
The effect of temperature for Eu(III) sorption onto cerium phosphate (initial pH: 4, initial concentration: 100 mg L^-1^, sorbent amount: 0.05 g, contact time: 120 min).

The effect of temperature on Eu(III) sorption was examined at 303, 313, 323 and 333 K. Figure 6 illustrates the variation of Q and K_D_ values as a function of temperature. Eu(III) uptake capacity slightly increases from 27.06 to 31.39 mg g^-1^ with the increase in temperature from 303 to 333 K. Similarly, a slight increase in K_D_ values from 493.4 to 657.0 mL g^-1^ is observed in the same region.

Thermodynamic data provides information about the spontaneity of the sorption process. The values of ΔH°, ΔS° and ΔG° were estimated using Eqs. (13), (14) and (15) [36]:

(13)KD=C0-CeCexVm

(14)lnKD=ΔS0R-ΔH0RT

(15)ΔG0=ΔH0-TΔS0

In the equations above, K_D_ is the thermodynamic equilibrium constant (mL g^-1^), R is the universal gas constant (8.314 J mol^-1^K^-1^), T is the temperature (K), V is the solution volume (mL) and mis the mass (g) of the sorbent. ΔH° and ΔS° were calculated from the slope and intercept of the plot between InK_D_ and 1/T.

Thermodynamic parameters for Eu(III) sorption are given in Table 6. The negative values of ΔG° obtained at 303, 313, 323, and 333 K point out the spontaneity of the Eu(III) sorption process.

**Table 6:** Thermodynamic parameters for Eu(III) sorption onto cerium phosphate.

Temperature (K)	ΔH° (kJ mol^-1^)	ΔS° (kJ mol^-1^K^-1^)	ΔG° (kJ mol^-1^)
303	7.35	0.08	− 16.89
313			− 17.69
323			− 18.49
333			− 19.29

The positive value of ΔH° suggests that Eu(III) sorption is an endothermic process. The positive value of ΔS° indicates the increase in randomness at the solid-liquid interface during sorption. In general, values of ΔG° for physisorption are up to − 20 kJ mol^-1^ while values between − 80 and − 400 kJ mol^-1^ indicate chemisorption mechanism [37]. The values of ΔGo obtained in this study are within the ranges of physisorption mechanism.

#### 3.4.3. Kinetic studies

Kinetic experiments were conducted between 5 and 360 min of contact time. The experimental kinetic data were evaluated using pseudo-first and pseudo-second-order kinetic models. Pseudo-first order model is expressed as:

(16)dqtdt=k1(qe-qt)

The integrated form of the equation is:

(17)ln(qe-qt)=lnqe-k1t

where k_1_ is the first-order rate constant (min^-1^), q_t_ and q_e_ are the amount of metal ion adsorbed (mg g^-1^) at time t and equilibrium, respectively [38,39]. k1 and qe can be obtained from the slope and the intercept of the plot.

Pseudo-first order kinetic model is not suitable to describe the sorption of Eu(III), because the R^2^ value is relatively low and the calculated q_e_ values are not per the experimental data.

The pseudo-second order model [40] is based on sorption capacity of sorbent and it is given by Eq. (18):

(18)dqtdt=k2(qe-qt)

where k_2_ is the second order rate constant (g mg^-1^min^-1^). Integrated linear form of the equation is expressed as:

(19)tqt=1k2qe2+tqe

**Figure 7:**
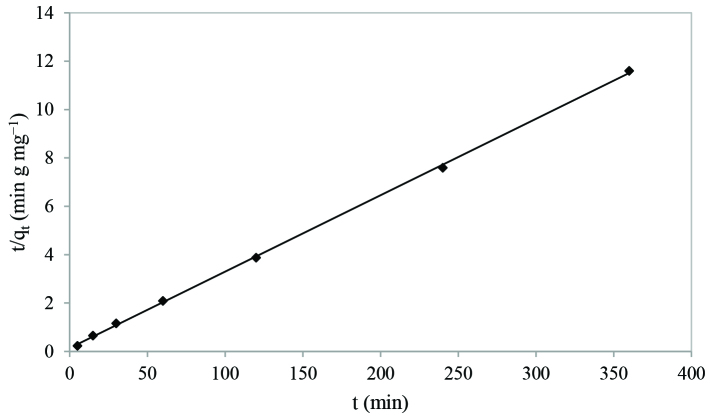
Pseudo-second order kinetic plot.

The kinetic plot of t/qt versus t for Eu(III) sorption is presented in Figure 7. q_e_ and k_2_ can be calculated from the slope and the intercept of the plot.

The pseudo-second-order model parameters q_e_, k_2_, and R^2^ are provided in Table 7. Depending on R^2^ values, the pseudo-second-order model suggests a better fit to experimental data.

**Table 7:** Kinetic model parameters for Eu(III) sorption.

Kinetic model	k_1_(min^-1^)	q_e_ (mg g^-1^)	R2
Pseudo-first order	0.008	6.15	0.72
	k_2_ (g mg min^-1^)	q_e_ (mg g^-1^)	R^2^
Pseudo-second order	0.007	31.65	0.99

## 4. Conclusion

In this study, amorphous cerium phosphate was synthesized via a one-step method. Characterization studies were performed by XRD, SEM, FTIR, and thermal analyses. The relation between independent variables (initial pH, contact time, sorbent amount) and Eu(III) sorption capacity (Q) was investigated by Box-Behnken design. The effect of initial pH and sorbent amount on Eu(III) sorption was found statistically significant by the evaluation of regression analyses and ANOVA. The correlation coefficient (R^2^ = 0.97) points out the good agreement between actual and predicted Q values. The F value of the model was found to be 15.93 and it shows the significance of the model. 3D response surface plots were described to evaluate the dual effects of variables. The maximum Eu(III) sorption capacity of cerium phosphate was obtained as 42.14 mg g^-1^ at initial pH 6, contact time of 180 min, and sorbent amount of 0.05 g.

Langmuir model is the best to describe the sorption of Eu(III) and monolayer Eu(III) sorption capacity was found to be 30.40 mg g^-1^. The positive value of ΔH° expresses the endothermic character of sorption. A decrease in ΔG° values with increasing temperature supports the assumption that the sorption process is spontaneous. Based on the value of R^2^, the pseudo-second-order model was best to fit the kinetic data.

Overall results point out that amorphous cerium phosphate is an easily prepared sorbent capable of removing Eu(III) ions from a slightly acidic aqueous solution.
